# Oxidative Stress-Induced DNA Damage Response Pathways in Aortic Disease: Implications for Inflammation and Vascular Degeneration

**DOI:** 10.3390/ijms27041855

**Published:** 2026-02-14

**Authors:** Sebastian Krych, Julia Gniewek, Marek Kolbowicz, Maria Adamczyk, Tomasz Hrapkowicz, Paweł Kowalczyk

**Affiliations:** 1 Department of Cardiac, Vascular and Endovascular Surgery and Transplantology, School of Medical Sciences in Zabrze, Medical University of Silesia, Marii Skłodowskiej-Curie 9, 41-800 Zabrze, Poland; thrapkowicz@sum.edu.pl; 2 Student’s Scientific Society, Department of Cardiac, Vascular and Endovascular Surgery and Transplantology, School of Medical Sciences in Zabrze, Medical University of Silesia, Marii Skłodowskiej-Curie 9, 41-800 Zabrze, Poland; julia.m.gniewek@gmail.com; 3 Institute of Physical Culture Sciences, University of Szczecin, Piastow 40b/6, 71-065 Szczecin, Poland; marek.kolbowicz@usz.edu.pl; 4 Institute of Spatial Management and Socio-Economic Geography, University of Szczecin, Mickiewicza 64, 71-101 Szczecin, Poland; maria.adamczyk@usz.edu.pl; 5 Department of Animal Nutrition, The Kielanowski Institute of Animal Physiology and Nutrition, Polish Academy of Sciences, Instytucka 3, 05-100 Jabłonna, Poland

**Keywords:** aortopathies, oxidative stress, DNA damage response, DNA repair, base excision repair, vascular smooth muscle cells, inflammation, cellular senescence, arteritis

## Abstract

Aortic diseases, including thoracic and abdominal aneurysms as well as aortic dissections, represent life-threatening vascular disorders characterized by progressive wall degeneration and inflammation. Increasing evidence indicates that oxidative stress is a central driver of aortic pathology through the induction of DNA damage in vascular smooth muscle cells and endothelial cells. Oxidative DNA lesions activate the DNA damage response (DDR), a highly coordinated network of damage sensors, signaling kinases, and repair effectors that determines cell fate decisions such as DNA repair, apoptosis, or cellular senescence. In aortic tissue, persistent or dysregulated DDR signaling contributes to chronic inflammation, extracellular matrix degradation, and loss of vascular integrity. Key molecular regulators, including base excision repair enzymes *OGG1* and *APE1*, as well as DDR mediators such as *ATM*, *ATR*, *p53*, *PARP*, and *NOTCH1*, integrate oxidative stress signals with pro-inflammatory and pro-degenerative pathways. Aberrant activation of these mechanisms promotes vascular smooth muscle cell VSMC phenotypic switching from contractile to synthetic phenotype, endothelial dysfunction, and senescence-associated secretory responses, thereby accelerating aortic wall weakening and aneurysm progression. This review highlights the mechanistic links between oxidative stress-induced DNA damage, DDR pathway activation, and vascular remodeling in aortopathies. A deeper understanding of these molecular interactions may uncover novel biomarkers and therapeutic targets aimed at limiting inflammation, preserving genomic stability, and preventing catastrophic aortic events. This work represents a narrative review and therefore has inherent limitations in terms of systematic literature search and selection.

## 1. Introduction: Aortopathies as Oxidative and Inflammatory Diseases

Aortopathies, including abdominal aortic aneurysms (AAAs), thoracic aortic aneurysms (TAAs), and aortic dissections, constitute a significant clinical cardiological and vascular problem, associated with high mortality rate due to rupture and sudden circulatory failure [1]. The pathogenesis of these conditions is complex and multifactorial, involving both hemodynamic factors and molecular mechanisms of vessel wall degeneration. Accumulating evidence indicates that oxidative stress and its consequences in the form of DNA damage play a key role in the development and progression of aortopathy through interaction with inflammatory processes, cell apoptosis, and extracellular matrix (ECM) degradation [1].

Oxidative stress is defined as an imbalance between the production of reactive oxygen species (ROS) and the antioxidant capacity of cells. Overproduction of ROS and reactive nitrogen species (RNS) leads to damage to proteins, lipids, and nucleic acids, including DNA, which has far-reaching consequences for the integrity of vascular cells and tissues [1,2]. In aortic tissue, ROS contribute to the degradation of vessel wall components, apoptosis of vascular smooth muscle, and activation of matrix metalloproteinases (MMPs), which contribute to weakening of the aortic structure and its pathological dilatation. Numerous studies in both humans and animal models confirm that ROS levels are elevated in the AAA wall and that chronic oxidative stress promotes its pathogenesis [1,3]. DNA damage induced by ROS includes, among others, oxidative base modifications such as 8-oxy-2′-deoxyguanosine (8-oxo-dG), single- and double-strand breaks, and other genotoxic changes that can lead to mutations, transcriptional abnormalities, and inappropriate repair responses [2,4]. Increased 8-oxo-dG immunoreactivity in endothelial and vascular smooth muscle cells has been observed in AAA patients, indicating increased oxidative DNA damage in aortic tissue compared to healthy controls [2,5]. The cellular response to DNA damage is supervised by evolutionarily conserved DNA Damage Response (DDR) mechanisms, which detect changes in the genetic material, arrest the cell cycle, and activate repair pathways such as base excision repair (BER) and double-strand break repair (DSBR) [2].

In the context of oxidative stress, these mechanisms become particularly important, as chronic exposure to ROS can overload repair systems, leading to their dysfunction and persistent DNA damage [3]. Persistent activation of the DDR can, in turn, trigger apoptotic pathways or induce cellular senescence, promoting a local proinflammatory environment and reducing the regenerative capacity of the vascular wall [3]. The pathological consequences of chronic oxidative stress and accumulated DNA damage are therefore twofold: they directly degrade the structure of the aortic wall through apoptosis and senescence of smooth muscle cells, and indirectly enhance inflammatory processes, promoting the development of vascular dysfunction and progression of aortopathy [4]. Furthermore, ROS modulate the expression of inflammatory genes through the activation of transcription factors such as NF-κB, further exacerbating inflammation within the aortic wall [4]. In light of these mechanisms, integrating the oxidative stress process with the DDR provides a promising interpretive framework for understanding the molecular basis of aortopathy. Analyzing the interactions between ROS, DNA damage, DDR, and inflammation may not only provide insight into the pathogenesis of these diseases but also identify potential therapeutic targets [4,5]. For example, DDR components, such as *ATM/ATR* kinases, *PARP1*, and BER proteins, may be targets for pharmacological modulation, which limits the progression of genetic damage and promotes genomic stability in vascular cells [5]. In summary, the role of oxidative stress and associated DNA damage, as well as DDR mechanisms, in aortopathies is a promising and intensively studied area of research. Understanding how ROS lead to genomic damage and how this damage influences inflammatory processes and vessel wall remodeling is crucial for developing new diagnostic and therapeutic strategies for aortic diseases [1,2,3,4,5]. As a narrative review, this work is subject to inherent limitations related to the absence of a fully systematic literature search and selection strategy.

**II. Pathogenic mechanisms: ROS, ECM degradation, VSMC dysfunction, and Notch/TGF-β alterations (Section 2 and Section 3).**

## 2. Oxidative DNA Damage in the Aortic Wall

### 2.1. Oxidative Stress as a Central Driver of Aortic Wall Degeneration

The aortic wall is particularly susceptible to oxidative stress due to its constant exposure to high hemodynamic pressure, pulsatile blood flow, and the presence of numerous sources of reactive oxygen species (ROS). Under physiological conditions, ROS serve a signaling function, regulating processes such as vascular smooth muscle cell (VSMC) proliferation and endothelial function. However, in aortopathies, their excessive production occurs, leading to pathological structural and molecular consequences.

### 2.2. Sources of ROS in the Aortic Wall

One of the main sources of ROS in aortic tissue are enzymes from the NADPH oxidase (NOX) family, particularly the *NOX1*, *NOX2*, and *NOX4* isoforms, whose expression is increased in aortic aneurysms. Studies have shown that *NOX* activation promotes elastin degradation, activation of matrix metalloproteinases (MMP-2 and MMP-9) and apoptosis of VSMCs, which weakens the structure of the vessel wall and promotes its dilation [6].

Mitochondria are also a significant source of ROS, whose dysfunction leads to increased electron leakage and superoxide anion generation. Reduced respiratory chain efficiency and accumulated mitochondrial DNA (mtDNA) damage are observed in VSMCs and endothelial cells derived from aortic aneurysms, promoting a vicious cycle of oxidative stress and cellular degeneration [7]. Furthermore, infiltrating inflammatory cells, such as macrophages and neutrophils, generate ROS through the activation of myeloperoxidase oxidase (MPO) and oxidative enzymes, exacerbating local oxidative stress and aortic tissue damage [8].

### 2.3. Consequences of Oxidative Stress on the Integrity of the Aortic Wall

Excessive ROS production leads to the oxidation of membrane lipids, carbonylation of structural proteins, and oxidative DNA damage. In the context of aortopathy, the effects of oxidative stress on VSMCs, which are responsible for maintaining the mechanical stability of the aorta, are particularly important [9]. ROS induce a phenotypic modulation of VSMCs from contractile to synthetic, which is associated with reduced expression of contractile proteins and increased production of ECM-degrading enzymes [9]. Furthermore, oxidative stress promotes the activation of programmed cell death and senescence, leading to the loss of functional VSMCs. Senescent cells secrete numerous cytokines, chemokines, and proteases as part of the so-called senescence-associated secretory phenotype (SASP), which further exacerbates inflammation and aortic wall remodeling [10].

### 2.4. Oxidative DNA Damage in Aortic Cells

DNA is one of the main targets of ROS, and oxidative modifications of nitrogenous bases constitute frequent and biologically relevant genomic damage. The best-known marker of oxidative DNA damage is 8-oxo-7,8-dihydroguanine (8-oxoG), the accumulation of which has been observed in aortic cells from patients with AAA and TAA. These changes affect both nuclear and mitochondrial DNA, with mtDNA damage appearing particularly severe due to limited mitochondrial protective mechanisms [11]. ROS-induced DNA damage can lead to replication errors, genomic instability, and activation of the DDR. Under conditions of chronic oxidative stress, these repair mechanisms can become overwhelmed, resulting in the accumulation of unrepaired DNA damage and persistent activation of stress signals in vascular cells [11].

### 2.5. Oxidative Stress as a Trigger of DNA Damage Response in Aortopathies

DNA Damage Response (DDR) activation in response to oxidative DNA damage involves the activation of ATM and ATR kinases, histone H2AX (γ-H2AX) phosphorylation, and the recruitment of repair complexes [12]. Increased expression of DDR markers in VSMCs and endothelial cells has been demonstrated in aortopathies, which correlates with the severity of aortic wall degeneration and disease progression [12]. Chronic DDR activation can lead to paradoxical effects: on the one hand, it protects cells from the mutagenic effects of DNA damage, while on the other, it promotes the induction of senescence and inflammation, contributing to the progression of aortic pathology. In this context, the DDR represents a key link integrating oxidative stress, inflammatory response, and vascular degeneration [11,12].

## 3. DNA Repair Mechanisms with Emphasis on BER

### 3.1. DNA Repair Pathways in Aortopathies: Focus on Base Excision Repair

Oxidative DNA damage is one of the most common genotoxic changes in vascular cells and plays a significant role in the pathogenesis of aortopathy. Due to constant exposure to reactive oxygen species (ROS), vascular smooth muscle cells (VSMCs) and aortic endothelial cells are particularly dependent on efficiently functioning DNA repair mechanisms [3,4,5,6]. Among the numerous repair pathways, base excision repair (BER) is the primary mechanism responsible for the removal of oxidative modifications of nitrogenous bases and minor DNA damage induced by oxidative stress [3,4,5,6].

### 3.2. Base Excision Repair Contributes to the Cellular Defense Against Oxidative DNA Damage

The base excision repair (BER) pathway is the primary mechanism responsible for recognizing and repairing DNA lesions, including modified bases, apurinic/apyrimidinic (AP) sites, and single-strand breaks [7,8,9,10,11]. This process is initiated by DNA glycosylases, which selectively recognize damaged bases and catalyze their removal [12]. Under conditions of oxidative stress, 8-oxoguanine DNA glycosylase 1 (OGG1) plays a central role by excising 8-oxo-7,8-dihydroguanine (8-oxoG), one of the most mutagenic products of oxidative DNA damage [13]. Removal of the damaged base generates an AP site, which is subsequently processed by apurinic/apyrimidinic endonuclease 1 (APE1). APE1 cleaves the DNA strand at the AP site, allowing recruitment of downstream repair enzymes, including DNA polymerase β and DNA ligase. Efficient execution of these steps is critical for maintaining genomic integrity, as accumulation of AP sites or incomplete repair can lead to DNA strand breaks, genomic instability, and cellular dysfunction [14].

OGG1 not only protects genomic and mitochondrial DNA from oxidative damage but also modulates vascular cell function and extracellular matrix homeostasis. Deficiency of OGG1 results in accumulation of oxidative lesions, persistent activation of the DNA damage response, endothelial and vascular smooth muscle cell (VSMC) dysfunction, induction of cellular senescence, and reduced expression of lysyl oxidase (LOX), collectively promoting vascular stiffening and susceptibility to aortic aneurysms [6,14,15,16,17,18,19,20,21,22,23,24,25,26,27,28,29,30,31,32,33,34,35,36,37,38,39,40,41]. Similarly, other DNA repair enzymes, including methylpurine DNA glycosylase (MPG) and O6-alkylguanine DNA alkyltransferase (ANPG/MGMT), repair alkylated and O6-alkylguanine lesions, thereby limiting chronic oxidative and inflammatory stress in vascular cells and indirectly influencing the progression of aortopathy [25,26,33,34,38,39,40,41].

Together, BER enzymes—including OGG1, MPG, and ANPG/MGMT—form a coordinated defense against genotoxic stress in vascular cells. Their activity not only preserves DNA integrity but also mitigates inflammatory signaling, maintains VSMC and endothelial cell function, and indirectly supports ECM stability through regulation of LOX and other matrix-regulatory proteins, highlighting their central role in preventing vascular ageing and aneurysm development.

### 3.3. Role of OGG1 and LOX in Vascular Homeostasis and Aortic Pathology

OGG1 (8-oxoguanine DNA glycosylase 1) is a central enzyme in the base excision repair (BER) pathway, responsible for recognizing and excising oxidatively damaged guanine bases (8-oxoG) generated by reactive oxygen species (ROS), thereby preserving genomic and mitochondrial DNA integrity under oxidative stress conditions [6,15,16,17,18,19,20,21,22]. Beyond its canonical role in DNA repair, OGG1 also modulates inflammatory gene transcription through interactions with transcription factors such as NF-κB, linking oxidative DNA damage to vascular inflammation [13,14,15]. In vascular smooth muscle cells (VSMCs) and endothelial cells, OGG1 deficiency leads to accumulation of oxidative DNA lesions, persistent activation of the DNA damage response, induction of cellular senescence, and impaired cell survival and repair capacity, ultimately compromising aortic wall homeostasis [13,14,15,16,23,24].

Mechanistically, OGG1 deficiency influences extracellular matrix (ECM) homeostasis in part through suppression of lysyl oxidase (LOX), a copper-dependent extracellular amine oxidase essential for collagen and elastin cross-linking and for maintaining the mechanical stability of the aortic wall [24,25,26,27,28,29,30,31,32]. Reduced LOX expression results in impaired ECM cross-linking, enhanced matrix degradation, medial degeneration, and increased vascular stiffening, collectively contributing to aortic dilation and heightened susceptibility to thoracic aortic aneurysm formation [6,18,19,20,21,22,30,31,32]. Consequently, dysregulated expression of ECM-regulatory proteins, particularly LOX downregulation, promotes vascular ageing, compromises vascular wall integrity, and increases aneurysm risk.

Focusing specifically on LOX, experimental models and human genetic studies highlight its critical and independent role in preserving aortic integrity. Loss-of-function variants in LOX have been identified in families with thoracic aortic aneurysm and dissection (TAAD), demonstrating that impaired LOX activity directly diminishes collagen and elastin cross-linking, promotes medial degeneration, and markedly increases susceptibility to aortic dilation and rupture [30,31,32]. Experimental evidence further indicates that LOX deficiency accelerates elastic fiber fragmentation and aortic dilatation, particularly under conditions of increased hemodynamic stress, whereas restoration or maintenance of LOX activity preserves ECM integrity and confers protection against aneurysm formation [30,31,32].

Collectively, these findings delineate a coordinated molecular network governing aortic wall homeostasis, in which DNA repair enzymes (including OGG1, MPG, and ANPG) mitigate ROS-induced genotoxic stress. Modulation of inflammatory signaling, cyclooxygenase (COX) enzymes regulate prostaglandin-mediated inflammatory responses that can exacerbate ECM degradation and vascular smooth muscle cell (VSMC) dysfunction, and lysyl oxidase (LOX) ensures proper extracellular matrix cross-linking essential for aortic mechanical resilience. Together, OGG1 and LOX represent complementary mechanisms preserving vascular integrity: OGG1 limits oxidative DNA damage and inflammatory activation, whereas LOX maintains ECM structure and tensile strength. Dysregulation of either pathway—or their combined impairment—synergistically promotes ROS accumulation, ECM destabilization, VSMC dysfunction, and progressive aortic wall weakening, thereby accelerating vascular ageing and increasing susceptibility to aneurysm formation and progression, providing a unified mechanistic framework linking oxidative genotoxic stress, inflammatory remodeling, and ECM instability in aortopathy [30,31,32].

### 3.4. Role of Other DNA Repair Enzymes and Cyclooxygenases COX-1 and COX-2 in Aortopathy

The integrity and function of the aortic wall depend not only on classical cardiovascular signaling pathways, such as transforming growth factor-β (TGF-β), angiotensin II (Ang II), and NOTCH signaling, but also on efficient DNA damage repair mechanisms, tightly regulated inflammatory responses, and preservation of extracellular matrix (ECM) architecture. Increasing evidence indicates that perturbations in these complementary systems substantially contribute to vascular wall degeneration and the development of aortopathy.

Among these regulatory mechanisms, DNA base excision repair (BER) enzymes, particularly DNA glycosylases such as alkyladenine DNA glycosylase (ANPG, also known as MPG), play a critical role in maintaining genomic stability in vascular smooth muscle cells (VSMCs) and endothelial cells exposed to oxidative and alkylating stress [13,14,15,16,17]. ANPG initiates BER by recognizing and excising a wide range of alkylated and deaminated DNA bases, thereby preventing mutagenesis, persistent activation of the DNA damage response, and premature cellular senescence. Impaired ANPG activity results in accumulation of DNA lesions, sustained oxidative stress, and chronic low-grade inflammation, which collectively promote VSMC dysfunction, apoptosis-hallmark features of aneurysmal degeneration of the aortic wall [13,14,15,16,17].

In addition to ANPG, other DNA glycosylases, including methylpurine DNA glycosylase (MPG), contribute to the repair of oxidized and alkylated purines, such as 1,N6-ethenoadenine and hypoxanthine, thereby modulating cellular responses to oxidative stress [25]. Although direct evidence linking MPG to aortic aneurysm formation remains limited, the cooperative activity of BER enzymes—including MPG, OGG1, and related glycosylases—prevents persistent oxidative DNA lesions that could otherwise promote VSMC dysfunction, apoptosis, and inflammatory gene expression, all of which are central contributors to aortopathy [25].

Furthermore, O6-alkylguanine DNA alkyltransferase (MGMT) participates in direct reversal repair of O6-alkylguanine lesions, preventing mutagenic base mispairing and limiting alkylation-induced genomic instability [26]. While the direct contribution of MGMT to aortopathy has not been specifically delineated, its protective role against chronic cellular stress and vascular inflammation suggests an important function in preserving aortic wall homeostasis [26].

In parallel with impaired DNA repair, chronic inflammatory signaling represents a major driver of aortic wall remodeling. Cyclooxygenase enzymes (COX-1 and COX-2), which catalyze the conversion of arachidonic acid into prostaglandins, serve as central mediators of vascular inflammation, neovascularization, and ECM turnover. In particular, COX-2 is markedly upregulated in human abdominal aortic aneurysm (AAA) tissue, predominantly within inflammatory infiltrates, macrophages, and activated VSMCs, where it promotes the production of pro-inflammatory prostaglandins that exacerbate ECM degradation and medial degeneration [27,28,29]. Experimental studies demonstrate that pharmacological inhibition or genetic deletion of COX-2 significantly attenuates aneurysm expansion, reduces inflammatory burden, and preserves ECM integrity, highlighting prostaglandin signaling as a critical mediator of aortic wall weakening and pathological remodeling [27,28,29].

Collectively, dysregulation of DNA repair pathways involving ANPG, MPG, OGG1, and MGMT, together with chronic inflammatory signaling mediated by COX enzymes, synergistically promotes oxidative stress, genomic instability, VSMC dysfunction, and ECM degradation. These interrelated processes accelerate vascular ageing and substantially increase susceptibility to thoracic and abdominal aortic aneurysm formation. Thus, DNA repair enzymes and COX-dependent inflammatory pathways represent critical, yet underappreciated, components of the molecular network governing aortic wall homeostasis and constitute promising therapeutic targets for limiting aneurysm progression [27,28,29].

### 3.5. Disruption of Molecular Mechanisms in Aortopathy

The structural integrity and functional resilience of the aortic wall rely on the coordinated interplay of multiple molecular processes, including classical signaling pathways (such as transforming growth factor-β [TGF-β], angiotensin II [Ang II], and NOTCH), efficient DNA damage repair, tightly regulated inflammatory responses, and proper extracellular matrix (ECM) cross-linking. Disruption of any of these processes—whether through persistent oxidative DNA damage with inadequate repair, chronic inflammation mediated by cyclooxygenase (COX) pathways, or defective ECM cross-linking due to lysyl oxidase (LOX) dysfunction—converges on a common pathophysiological outcome: weakening of the aortic wall and increased susceptibility to aneurysm formation and progression [17,18,23,24,29,30].

Persistent oxidative DNA lesions in vascular smooth muscle cells (VSMCs) and endothelial cells, when not adequately repaired by base excision repair enzymes such as OGG1, MPG, and ANPG, trigger chronic activation of the DNA damage response, promote cellular senescence, and impair repair capacity. These events compromise cellular resilience, exacerbate reactive oxygen species (ROS) accumulation, and link genotoxic stress to inflammatory signaling pathways, creating a feed-forward loop that undermines vascular homeostasis [17,18,23,24].

Simultaneously, chronic inflammatory signaling, particularly via COX-derived prostaglandins, amplifies ECM degradation and medial degeneration. Elevated COX-2 expression in aneurysmal aortic tissue promotes recruitment of inflammatory cells, VSMC dysfunction, and neovascularization, all of which contribute to weakening of the aortic wall and facilitation of aneurysm expansion [17,18,29]. Pharmacological inhibition of COX-2 in experimental models attenuates aneurysm progression, demonstrating the causal role of prostaglandin-mediated inflammation in aortic remodeling.

Proper ECM cross-linking, mediated primarily by LOX, is essential for maintaining mechanical strength and elasticity of the aortic wall. LOX deficiency, whether due to genetic loss-of-function variants or downregulation secondary to oxidative stress, impairs collagen and elastin cross-linking, promotes elastic fiber fragmentation, and accelerates medial degeneration, particularly under hemodynamic stress [30]. The combined impairment of LOX-mediated ECM stabilization with persistent DNA damage and inflammation synergistically amplifies aortic wall fragility.

Collectively, these interconnected mechanisms highlight the **multifactorial nature of aortopathy**, wherein genetic susceptibility, oxidative stress, chronic inflammation, and ECM instability converge to drive disease progression. Understanding this complex network provides critical insight into potential therapeutic targets, including interventions aimed at enhancing DNA repair capacity, modulating inflammatory signaling, and preserving ECM integrity, offering a comprehensive strategy for preventing or limiting aneurysm formation and progression [17,18,23,24,29,30].

### 3.6. Integrated Impact of Impaired DNA Repair, Inflammation, and ECM Dysfunction on Aortic Pathology

The integrity of the aortic wall depends on a finely tuned interplay between DNA repair mechanisms, inflammatory signaling, and extracellular matrix (ECM) maintenance. Disruption of any of these processes—persistent oxidative DNA damage due to deficient repair enzymes such as OGG1, MPG, or ANPG/MGMT, chronic inflammatory signaling mediated by cyclooxygenase-2 (COX-2), or defective ECM cross-linking resulting from LOX dysfunction—converges to compromise vascular wall stability and increase susceptibility to aneurysm formation and progression [27,28,29,30,31,32].

OGG1, MPG, and ANPG collectively safeguard genomic and mitochondrial DNA in vascular smooth muscle cells (VSMCs) and endothelial cells, limiting oxidative and alkylative stress and preventing activation of the DNA damage response, cellular senescence, and VSMC dysfunction. Concurrently, COX-2-driven prostaglandin synthesis amplifies inflammatory responses, promotes ECM degradation, and facilitates neovascularization within the aortic wall, as evidenced by its upregulation in aneurysmal tissue and attenuation of aneurysm progression following pharmacologic inhibition [27,28,29]. LOX ensures proper collagen and elastin cross-linking, maintaining the tensile strength and elasticity of the aortic wall; loss-of-function variants or reduced LOX activity accelerate medial degeneration, elastic fiber fragmentation, and aortic dilation, particularly under hemodynamic stress [30,31,32].

Together, these interrelated mechanisms illustrate a **synergistic network of vascular vulnerability**: impaired DNA repair increases ROS accumulation and cellular stress, COX-2-mediated inflammation exacerbates ECM breakdown and medial weakening, and LOX deficiency reduces the mechanical resilience of the aortic wall. The combined disruption of these pathways accelerates vascular ageing, promotes aneurysm formation, and enhances the risk of aortic dissection or rupture [27,28,29,30,31,32].

**Figure 1:**
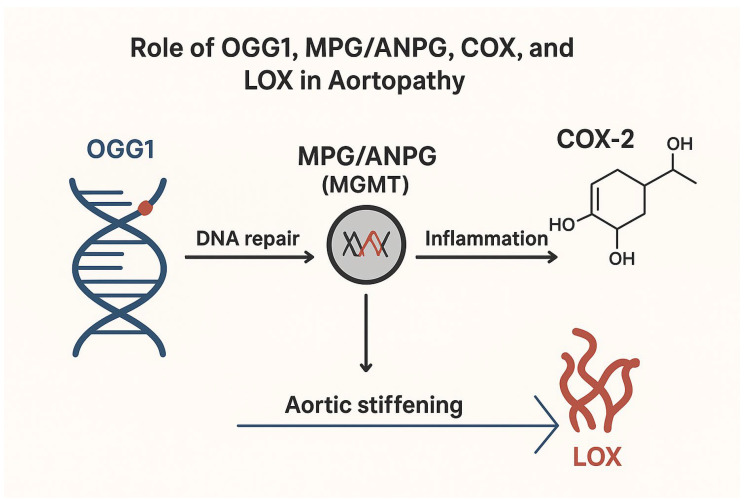
Schematic representation of the role of OGG1, MPG/ANPG, COX-2, and LOX in the development of aortopathy and aortic stiffening.

These insights underscore the **multifactorial nature of aortopathy**, highlighting that therapeutic strategies should not focus on a single pathway but rather target multiple interconnected mechanisms. Interventions aimed at enhancing DNA repair capacity, modulating inflammatory prostaglandin signaling, and preserving ECM cross-linking integrity represent promising avenues to maintain aortic wall homeostasis and limit aneurysm progression (Figure 1). By integrating the molecular contributions of OGG1, MPG/ANPG, COX-2, and LOX, this framework provides a unified mechanistic understanding of aortic wall vulnerability and the pathogenesis of aneurysmal disease [27,28,29,30,31,32].

The figure illustrates the proposed molecular mechanisms linking DNA damage repair, inflammatory signaling, and extracellular matrix remodeling in the pathogenesis of aortopathy. Oxidative DNA damage in vascular cells is recognized and repaired primarily by 8-oxoguanine DNA glycosylase 1 (OGG1), a key enzyme of the base excision repair (BER) pathway, which removes oxidized purine lesions from DNA. In parallel, N-methylpurine DNA glycosylase (MPG, also known as ANPG) and O6-methylguanine-DNA methyltransferase (MGMT) participate in the repair of alkylated DNA bases, thereby maintaining genomic stability. Dysregulation or excessive activation of these DNA repair mechanisms may contribute to persistent cellular stress responses.

DNA damage and defective repair are associated with activation of inflammatory pathways, including upregulation of cyclooxygenase-2 (COX-2), an inducible enzyme responsible for prostaglandin synthesis and amplification of vascular inflammation. Chronic inflammation promotes vascular remodeling and stimulates the expression and activity of lysyl oxidase (LOX), an enzyme involved in collagen and elastin cross-linking within the extracellular matrix. Increased LOX activity leads to enhanced cross-linking of structural proteins, resulting in increased aortic wall stiffness. Collectively, these processes contribute to progressive aortic stiffening and the development of aortopathy.

Arrows indicate the proposed functional relationships between DNA damage repair, inflammation, extracellular matrix remodeling, and vascular stiffening.

In addition to DNA repair genes, the role of the *NOTCH1* gene, which encodes one of the receptors involved in an evolutionarily conserved signaling pathway, is also crucial. The Notch gene plays a key role in the embryogenesis of the cardiovascular system, determining cell fate, and maintaining the homeostasis of endothelial and vascular smooth muscle cells.

### 3.7. The NOTCH 1 Gene

The *NOTCH1* gene (as an exemplar of DDR–genetic interactions) encodes the transmembrane receptor Notch1, a key component of the evolutionarily conserved Notch signaling pathway that regulates intercellular communication and cell fate determination. Ligand binding (Jagged or Delta-like ligands) on adjacent cells triggers proteolytic cleavage of the receptor and release of the Notch intracellular domain (NICD), which translocates into the nucleus and modulates transcription of canonical target genes, including HES and HEY family members. Through this mechanism, *NOTCH1* signaling controls vascular smooth muscle cell (VSMC) proliferation, differentiation, survival, and phenotypic stability, thereby maintaining vascular wall homeostasis [38,39,40,41].

Pathogenic variants in *NOTCH1* have been strongly associated with congenital heart defects, particularly bicuspid aortic valve (BAV), and with the development of ascending aortic aneurysms (AscAAs) [2]. Experimental models demonstrate that Notch1 deficiency leads to medial degeneration, abnormal VSMC phenotype switching, and progressive aortopathy. Importantly, *NOTCH1* mutations have also been identified in patients with thoracic aortic aneurysms (TAAs) and tricuspid aortic valves, indicating that NOTCH1 haploinsufficiency can contribute to aneurysm formation independently of valve morphology [38,42,43,44,45,46,47,48,49,50,51,52,53,54,55,56,57,58,59,60,61,62,63,64,65,66,67,68,69,70,71,72,73,74].

At the molecular level, *NOTCH1* signaling interacts closely with transforming growth factor-β (TGF-β) pathways and regulates extracellular matrix (ECM) turnover through modulation of matrix metalloproteinases, particularly *MMP-2* and *MMP-9*. Reduced *NOTCH1* activity is associated with increased *MMP* expression, enhanced elastin and collagen degradation, and weakening of the aortic wall. Additionally, *NOTCH1* contributes to DNA damage response and cellular stress adaptation, linking genomic instability to inflammatory and remodeling processes. Collectively, dysregulation of NOTCH1 signaling integrates genetic susceptibility, altered cellular behavior, and ECM degradation, thereby playing a central role in the pathogenesis of aortopathy [38,44,45,46,47,48,49,50,51,52,53,54,55,56,57,58,59,60,61,62,63,64,65,66,67,68,69,70,71,72,73,74,75,76,77,78,79,80,81,82,83,84,85,86,87,88,89,90,91,92,93,94,95,96,97,98,99].

#### 3.7.1. Jagged (JAG1/2) and (DLL1/3/4) Families

Upon binding to ligands from the Jagged *(JAG1/2*) or Delta-like (*DLL1/3/4*) families, the *NOTCH1* receptor undergoes proteolytic release of its intracellular domain (NICD), which translocates to the nucleus and regulates the expression of target genes, such as HES1 and other transcription factors involved in vascular wall cell differentiation [38,42,43,44,45,46,47,48,49,50,51]. *NOTCH 1* mutations in humans are associated with bicuspid congenital aortic valve disease (BAV) and with an increased risk of developing ascending aortic aneurysms. Recent genetic studies also show that *NOTCH 1* haploinsufficiency—i.e., loss of one functional copy of the gene—may be a pathogenic mechanism leading directly to nonsyndromic thoracic aortic aneurysms, even in patients with tricuspid valves, suggesting a direct involvement of this gene in the pathogenesis of aortopathy [38,42,43,44,45,46,47,48,49,50,51]. At the molecular level, *NOTCH 1* influences the structure of the aortic wall by regulating the phenotype of vascular smooth muscle cells (VSMCs) and their interaction with the extracellular matrix (ECM). Under normal conditions, Notch activation promotes the expression of genes that preserve VSMC contractile markers, while its loss results in increased apoptosis and impaired differentiation, as observed in aortic tissues from patients with BAV-associated aortopathy [38,42,43,44,45,46,47,48,49,50,51].

#### 3.7.2. VSMC Dysfunction

VSMC dysfunction leads to weakened vessel wall structure, which in turn predisposes to aortic dilatation and aneurysm formation. Another key aspect is the interaction of the Notch pathway with TGF-β, which is fundamental for vessel wall remodeling. Notch and TGFβ can co-regulate the expression of VSMC contractile genes through the interaction of Smads with Notch-dependent factors, but they can also antagonize each other. For example, Notch induces miR 145, which downregulates the expression of the TGFβ type II receptor, and TGFβ, in return can downregulate the expression of some Notch receptors, suggesting complex signaling regulation in the aortic wall [38,42,43,44,45,46,47,48,49,50,51]. This coupling regulates the balance between the contractile and relaxation phenotypes of VSMCs and plays a crucial role in maintaining vascular wall integrity. An important mechanism in the pathogenesis of aortopathy is the degradation of the extracellular matrix (ECM), catalyzed by enzymes such as *MMP 2* and *MMP 9*.

#### 3.7.3. Loss of Notch Signaling

Loss of Notch signaling can lead to the overexpression of these metalloproteinases, which promotes collagen and elastin degradation, weakening the vascular wall and facilitating aneurysm progression [38,42,43,44,45,46,47,48,49,50,51]. These interactions are in turn tightly coupled to TGF β signals, which through their cascade of *SMAD3-6* and *FBN1* genes influence the ECM balance and may further modulate *MMP* activity [51,52,53,54,55,56,57,58,59,60,61,62,63,64,65,66,67,68,69,70,71,72,73,74,75,76]. *NOTCH* signaling regulates cell fate, proliferation, and differentiation in both VSMCs and endothelial cells of the aortic wall. Activation occurs when *NOTCH* receptors, such as *NOTCH 1*, interact with ligands on adjacent cells, triggering proteolytic cleavage and release of the Notch intracellular domain (NICD), which enters the nucleus to modulate gene expression. Haploinsufficiency or mutations in *NOTCH 1* are associated with bicuspid aortic valve (BAV) and related aortopathies, including ascending aortic aneurysms. Notably, *NOTCH 1* signaling interacts with TGF-β pathways in VSMCs, influencing ECM production and cellular proliferation, which are critical for maintaining aortic wall integrity [64,74,75,76,77,78,79,80].

#### 3.7.4. The FBN1 Gene

The *FBN1* gene encodes the large extracellular matrix glycoprotein *fibrillin-1*, which is the core structural component of microfibrils providing mechanical support to connective tissues, both elastic and non-elastic. Its structure contains multiple epidermal growth factor (EGF)-like repeats necessary for fiber polymerization and tensile strength [76]. Mutations in *FBN1* are classically associated with *Marfan syndrome*, an autosomal dominant connective tissue disorder presenting with skeletal overgrowth, cardiovascular abnormalities (especially aortic aneurysms), and ocular manifestations. The phenotype arises from both structural defects in connective tissue and dysregulation of TGF-β and BMP signaling due to altered fibrillin-1 interactions, exacerbating pathological processes in tissues [76]. Functional studies, including zebrafish models, have confirmed the deleterious effects of novel pathogenic *FBN1* variants on microfibril formation and tissue integrity [77].

#### 3.7.5. The SMAD6 Gene

**Figure 2:**
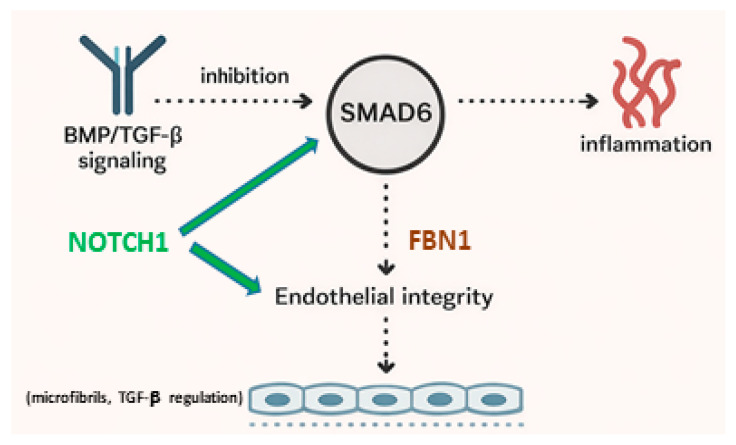
Schematic overview of key cell signaling pathways involved in the regulation of vascular homeostasis and aortopathy: TGF-β, *NOTCH*, and angiotensin II (Ang II).

The *SMAD6* gene belongs to the Smad family and functions as an intracellular inhibitor of BMP (bone morphogenetic protein) signalling, a subfamily within the larger TGF-β superfamily. *SMAD6* competes with SMAD4 for binding to receptors or receptor-regulated Smads (such as *SMAD1/5/8*), preventing the formation of transcriptionally active complexes and thereby suppressing BMP-dependent gene activation. This regulation balances proliferation, differentiation, and apoptosis during development [60,61,62,63,64,65,66,67,68,69,70,71,72,73,74,75]. Clinically, pathogenic variants in *SMAD6* are associated with a spectrum of congenital malformations, including congenital heart defects (e.g., BAV and aortic aneurysms), left ventricular outflow tract malformations, and skeletal anomalies, highlighting its critical role in embryonic tissue patterning [60]. Functional studies have demonstrated that *SMAD6* deficiency disrupts BMP inhibition, leading to severe aortic valve calcification and thoracic aortic aneurysms in patients, and also acts downstream of Notch1 in endothelial cells to maintain vascular homeostasis through regulation of genes involved in cell adhesion and vessel wall architecture [72,73,74,75] (Figure 2).

The figure illustrates the major intracellular signaling cascades activated by transforming growth factor-β (TGF-β), *NOTCH* receptors, and angiotensin II (Ang II), which collectively regulate vascular development, smooth muscle cell phenotype, extracellular matrix turnover, inflammation, and remodeling of the aortic wall. Binding of TGF-β to its type I and type II serine/threonine kinase receptors leads to phosphorylation and activation of receptor-regulated *SMAD* proteins (*SMAD2* and *SMAD3*), which form complexes with *SMAD4* and translocate into the nucleus to regulate transcription of genes involved in extracellular matrix synthesis, fibrosis, and cell differentiation. In parallel, non-canonical TGF-β signaling pathways, including mitogen-activated protein kinases (MAPKs), phosphatidylinositol-3-kinase/protein kinase B (PI3K/AKT), and Rho-like GTPases, modulate cytoskeletal dynamics, cell migration, and survival.

*NOTCH* signaling is initiated by ligand binding (e.g., Jagged and Delta-like ligands) to *NOTCH* receptors, resulting in proteolytic cleavage and release of the *NOTCH* intracellular domain (NICD). The NICD translocates into the nucleus, where it regulates transcription of genes controlling vascular smooth muscle cell differentiation, proliferation, and apoptosis, thereby contributing to the maintenance of vascular structure and function.

Angiotensin II signaling via the angiotensin II type 1 receptor (AT1R) activates multiple downstream pathways, including MAPKs, nuclear factor-κB (NF-κB), and reactive oxygen species (ROS) production, promoting inflammation, oxidative stress, vasoconstriction, and extracellular matrix remodeling. Crosstalk among TGF-β, *NOTCH*, and Ang II signaling pathways integrates mechanical and biochemical stimuli, shaping adaptive and maladaptive remodeling of the aortic wall. Dysregulation of these interconnected signaling networks contributes to pathological vascular remodeling, aortic stiffening, and the progression of aortopathy.

Arrows indicate activation or regulatory interactions among signaling components, highlighting the complex network governing aortic wall homeostasis and disease development.

Aortopathy, including thoracic aortic aneurysm (TAA) and aortic dissection, arises from complex interactions among cellular signaling pathways that regulate vascular smooth muscle cell (VSMC) function, extracellular matrix (ECM) integrity, and endothelial homeostasis. Among these pathways, TGF-β (transforming growth factor-beta), *NOTCH*, and angiotensin II (Ang II) play central and interconnected roles in maintaining aortic wall structure and function [33,34,35,38,43,44,45,46,47,48,49,50].

#### 3.7.6. TGF-β Signalling

TGF-β signaling is essential for the regulation of ECM composition and VSMC differentiation. In the canonical pathway, TGF-β ligands bind to type I and type II receptors, leading to phosphorylation of receptor-regulated SMADs (*SMAD2/3*), which then form complexes with *SMAD4* and translocate to the nucleus to regulate gene transcription. Dysregulation of this pathway, often caused by mutations in genes such as *FBN1*, *SMAD3*, or *SMAD6*, results in impaired ECM remodeling, loss of aortic elasticity, and increased susceptibility to aneurysm formation. For example, in Marfan syndrome, defective fibrillin-1 fails to sequester TGF-β properly, leading to excessive signaling that drives progressive aortic dilation [48,70,71,72,73,74,75,76,77].

#### 3.7.7. Angiotensin II (Ang II)

Angiotensin II (Ang II), a major effector of the renin–angiotensin system, promotes VSMC proliferation, oxidative stress, inflammation, and ECM remodeling through activation of AT1 receptors. Chronic Ang II signaling exacerbates medial thickening, elastic fiber degradation, and inflammatory responses, contributing to aortic dilation and aneurysm formation. Ang II also upregulates TGF-β activity and interacts with *NOTCH* pathways, creating a feed-forward loop that amplifies pro-aneurysmal mechanisms [33,34]. Together, these pathways form an integrated network in which dysregulation of one component can destabilize the aortic wall. Excessive TGF-β activity due to ECM defects, altered *NOTCH1* signaling, or heightened Ang II stimulation leads to medial degeneration, VSMC apoptosis, and loss of elastic fiber integrity, ultimately predisposing the aorta to aneurysm formation and dissection. Understanding these molecular interactions has significant therapeutic implications, as interventions targeting TGF-β signaling, AT1 receptors, or *NOTCH* modulation are being explored to prevent the progression of aortopathies [37]. In the context of microRNA (miRNA) and epigenetic regulation of Notch signaling, several miRNAs modulate the expression of components of this pathway and genes associated with aortopathy. MiR 145 is one of the regulators of the VSMC phenotype and a negative regulator of the TGFβ receptor, thus influencing both Notch and TGFβ signaling [56,57,58,59,60,61,62,63,64,65]. Other miRNAs, such as miR 26a, regulate TGFβ/Smad signaling, contributing to modulating VSMC proliferation and apoptosis, which is important in aortic remodeling [56,57,58,59,60,61,62,63,64,65]. Although specific miRNAs only partially directly influence *NOTCH1*, the overall miRNA expression profile in aortopathy reflects cellular dysfunctions relevant to disease progression [56,57,58,59,60,61,62,63,64,65]. Additionally, emerging data suggest that mitochondrial dysfunction may be an important pathogenetic mechanism in *NOTCH1*-associated aortopathy, where its deficiency leads to loss of mitochondrial function, which exacerbates aortic wall weakening and accelerates aneurysm progression in animal models [56,57,58,59,60,61,62,63,64,65,66,67,68,69,70].

In summary, *NOTCH1* and other genes play a multifaceted role in aortopathy by:

- Regulating VSMC differentiation and survival, where its disruption promotes apoptosis and the synthetic phenotype of smooth muscle cells, weakening the aortic wall.
- Modulating TGF-β signaling, which regulates the balance between ECM remodeling and stability.
- Influencing the expression of ECM metalloproteinases (MMP 2/MMP 9), which degrade ECM components.
- Interacting with miRNAs and epigenetic elements that modulate both Notch and other signaling pathways in aortopathy.
- Participating in mitochondrial mechanisms that influence the metabolic homeostasis of vessel wall cells. Such phenomena make *NOTCH1* and its related signaling elements promising molecular markers of aortopathy risk and potential therapeutic targets, although their clinical penetrance is variable and modulated by interactions with other genes, environmental and epigenetic factors [66,67,68,69,70] (Table 1).

**Figure 3:**
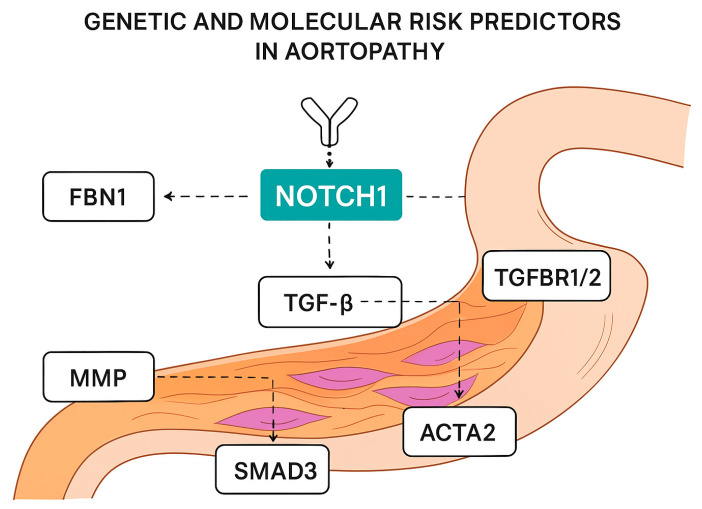
Genetic and molecular risk predictors associated with NOTCH1 signaling in the pathogenesis and progression of thoracic aortopathy.

Key observations:

- *NOTCH1* acts primarily at the level of VSMC differentiation and phenotype and endothelial signaling, modulating ECM remodeling via MMPs.
- *FBN1*, *TGFBR1/2*, and *SMAD3* have a major impact on ECM structure and TGFβ signal transduction, which directly modulates aortic elasticity.
- *ACTA2* and *MYH11* influence VSMC mechanics and vessel wall contractility.
- Many of these genes interact with *NOTCH1*, for example, TGFβ indirectly modulating the Notch pathway and vice versa, highlighting the complexity of genetic predictors of aortopathy risk (Figure 3).

The figure summarizes the key genetic variants, molecular pathways, and downstream cellular mechanisms linked to *NOTCH1* dysfunction that contribute to the development and progression of thoracic aortic disease. Pathogenic and likely pathogenic variants in the *NOTCH1* gene lead to altered receptor activation, impaired proteolytic cleavage, and reduced generation of the *NOTCH* intracellular domain (NICD), resulting in dysregulated transcription of canonical target genes, including members of the HES and HEY families. This disruption affects vascular smooth muscle cell (VSMC) differentiation, proliferation, and survival, as well as endothelial cell function, ultimately compromising aortic wall integrity.

At the molecular level, defective *NOTCH1* signaling promotes increased activity of matrix metalloproteinases (*MMP-2* and *MMP-9*), enhanced extracellular matrix (ECM) degradation, and impaired elastin fiber organization, leading to weakening of the aortic media. Concurrently, altered *NOTCH1* signaling interacts with transforming growth factor-β (TGF-β) pathways and regulatory microRNAs, such as miR-145 and miR-34a, further modulating VSMC phenotype switching, inflammatory responses, and ECM remodeling.

The figure also highlights the contribution of oxidative stress, inflammatory mediators, and mechanotransduction signals to NOTCH1-dependent vascular remodeling. These combined genetic and molecular alterations increase susceptibility to bicuspid aortic valve (BAV), ascending aortic aneurysm formation, and aortic dissection. Arrows indicate the direction of signaling events and regulatory interactions, illustrating the integrated network of pathways that collectively determine disease risk and clinical phenotype.

Mutations or rare variants in genes such as *NOTCH 1* have been implicated in BAV for a long time. Recent data suggest that such mutations may also contribute to associated aortopathy: experimental models show that haploinsufficiency of *NOTCH 1* can be sufficient to cause ascending aortic aneurysm (AscAA) even in absence of overt BAV, highlighting a direct role of *NOTCH 1* signaling in vascular wall integrity [38,40,41,44,45,65]. Similarly, as noted above, loss-of-function variants in *SMAD6* have been significantly associated with BAV + TAA in human cohorts [36]. Variants in other ECM-related genes (e.g., *FBN1*, coding for fibrillin-1) have been proposed to contribute to aortic wall susceptibility, possibly via dysregulation of ECM assembly and growth-factor sequestration (e.g., TGF-β binding)—similarly to mechanisms seen in syndromic aortopathies such as Marfan syndrome [36,41,48,70,71]. Epigenetic and transcriptomic studies suggest that BAV aortopathy is heterogeneous: different “phenotypes” of aortic wall disease may exist, depending on specific gene variants, alternative splicing patterns (e.g., of fibronectin), and differential activation of signaling pathways (TGF-β/BMP/Notch), which may in turn influence the risk and progression of aneurysm [20,36,55]. Notably, some studies propose that the aortopathy in BAV may not be a uniform entity—but rather a spectrum of remodeling patterns, varying between individuals depending on genetic background and molecular milieu [32,33,34,35,36,37,38,39,40,41,42,43,44,45,46,47,48,49,50,51,52,53,54,55,56,57,58,59,60,61,62,63,64,65,66,67,68,69,70,71,72,73,74,75].

#### 3.7.8. Clinical Significance of Molecular Mechanisms

**Figure 4:**
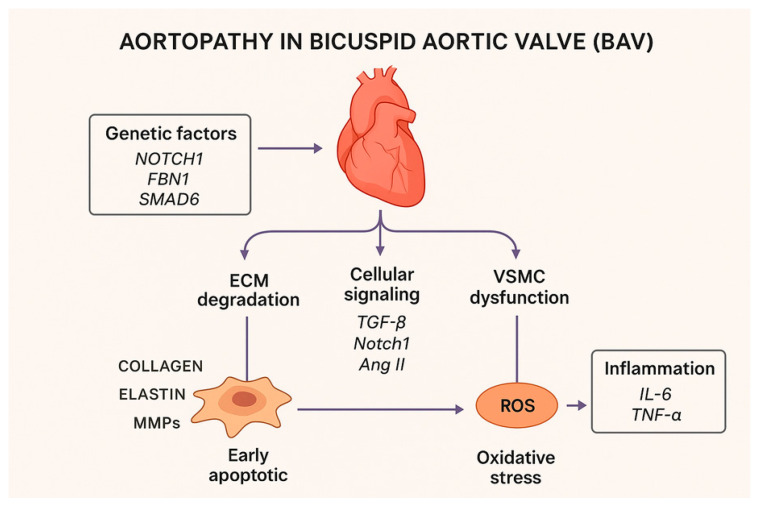
Pathophysiological mechanisms of aortopathy associated with bicuspid aortic valve (BAV).

Understanding these molecular and genetic underpinnings helps to identify high-risk patients even when aortic dilatation is only mild or in early stage—for whom mere measurement of diameter may underestimate the risk. These insights open the door for targeted therapeutic strategies: for example, modulation of TGF-β/BMP signaling, inhibition of excessive MMP activity, or pharmacologic regulation of VSMC phenotype. Indeed, some authors have proposed that modulating TGF-β activity (or its downstream signaling) could be a therapeutic avenue, although human data remain scarce [36]. Genetic testing (e.g., for *SMAD6*, *NOTCH 1*, *FBN1* variants)—combined with molecular profiling of aortic tissue—might in future allow personalized risk stratification and tailored surveillance or intervention strategies beyond standard size-based criteria. Recognizing that BAV aortopathy is not solely a consequence of abnormal flow dynamics but also involves inherent structural and molecular wall defects may shift our conceptual framework, improving prevention, monitoring, and potentially therapy [72,73,74,75,76,77] (Figure 4).

The figure illustrates the multifactorial mechanisms underlying the development and progression of aortopathy in patients with bicuspid aortic valve (BAV), integrating genetic predisposition, altered hemodynamic forces, cellular dysfunction, and extracellular matrix (ECM) remodeling. BAV is characterized by abnormal valve morphology, typically resulting from congenital fusion of two aortic valve cusps, which leads to disturbed blood flow patterns and abnormal wall shear stress within the ascending aorta. These altered hemodynamic conditions promote endothelial dysfunction, increased oxidative stress, and activation of inflammatory signaling pathways.

At the cellular level, vascular smooth muscle cells (VSMCs) exhibit from a contractile to a synthetic state, associated with reduced expression of contractile proteins, increased proliferation, and enhanced secretion of matrix metalloproteinases (MMPs), particularly MMP-2 and MMP-9. This shift contributes to excessive degradation of elastin and collagen fibers, weakening the medial layer of the aortic wall. In parallel, dysregulated signaling pathways, including transforming growth factor-β (TGF-β), NOTCH, and angiotensin II (Ang II), further modulate ECM turnover, inflammation, and vascular remodeling.

Genetic factors, such as pathogenic variants in NOTCH1 and other aortopathy-related genes, predispose individuals with BAV to abnormal aortic wall structure and impaired repair mechanisms. The combined effects of genetic susceptibility, chronic hemodynamic stress, and molecular dysregulation result in progressive aortic dilation, increased wall stiffness, and heightened risk of ascending aortic aneurysm formation and aortic dissection.

Arrows indicate the direction and interaction of mechanical, genetic, and molecular signals contributing to maladaptive remodeling of the aortic wall, highlighting the complex and dynamic processes driving BAV-associated aortopathy.

#### 3.7.9. *APE1* as a Dual-Function Protein in DNA Repair and Redox Signaling

*APE1* is a dual-function protein combining BER endonuclease activity with a role as a redox regulator of transcription factors. In addition to its key role in DNA repair, *APE1* modulates the activity of NF-κB, *AP-1*, and HIF-1α by maintaining their reduced state, capable of binding DNA. This property makes *APE1* an important hub integrating oxidative stress, DNA repair, and the inflammatory response [81]. Increased *APE1* expression is observed in vascular diseases, which may reflect an adaptive response to increased DNA damage. However, chronic *APE1* activation may paradoxically promote the persistence of inflammation through redox-dependent activation of proinflammatory genes. In aortopathies, such a feedback loop may lead to simultaneous exacerbation of inflammation and overload of DNA repair mechanisms, contributing to disease progression [82].

#### 3.7.10. PARP1 as a Sensor of DNA Strand Breaks and Mediator of Inflammation

Poly(ADP-ribose) polymerase 1 (*PARP1*) is one of the first proteins recruited to DNA strand breaks and plays a key role in coordinating DNA repair by modifying chromatin proteins and recruiting repair complexes. *PARP1* activation is particularly important in response to oxidative DNA damage leading to single-strand breaks. However, excessive or chronic *PARP1* activation can lead to NAD^+^ and ATP depletion, resulting in cellular dysfunction and cell death. Increased *PARP1* activity has been demonstrated in aortic tissue from patients with aneurysms, which correlates with increased inflammation and extracellular matrix degradation [83]. Importantly, *PARP1* is also a potent modulator of the inflammatory response, interacting with NF-κB and other transcription factors. In this way, PARP1 links DNA damage with inflammatory activation, creating a molecular basis for the progression of aortopathy under conditions of chronic oxidative stress.

#### 3.7.11. Impaired DNA Repair as a Driver of Vascular Degeneration

Dysfunction of BER and key proteins such as OGG1, APE1, and PARP1 leads to the accumulation of DNA damage, chronic activation of DDR, and induction of senescence, apoptosis, and phenotypic switching of vascular smooth muscle cells (VSMCs). This condition promotes the development of a proinflammatory microenvironment and structural weakening of the aortic wall [84,85,86,87,88]. In aortopathies, DNA repair dysfunction can therefore be viewed not only as a consequence of oxidative stress but also as an active driver of vascular degeneration. Understanding the molecular mechanisms regulating BER in aortic cells opens new therapeutic possibilities, including modulation of PARP1 activity, enhancement of OGG1 function, and targeting the redox-dependent functions of APE1 [84,85,86,87,88].

**IV. Integrated mechanisms: DNA damage → cGAS** **–STING → chronic inflammation (Section 4).**

## 4. Crosstalk Between DNA Damage Response, NF-κB Signaling, and cGAS–STING Pathway in Aortopathies

Accumulating evidence indicates that the cellular response to DNA damage extends beyond classical mechanisms of cell cycle arrest and genomic repair to include activation of the innate immune response and inflammatory processes. In the context of aortopathies, in which chronic oxidative stress leads to persistent DNA damage, the DNA Damage Response (DDR) plays a central role as an integrator of genotoxic, inflammatory, and degenerative signals [89]. Of particular importance is the functional coupling between the DDR, the NF-κB pathway, and the cytoplasmic DNA sensing system cGAS–STING, which together shape the proinflammatory microenvironment of the aortic wall [89].

### 4.1. DDR as an Upstream Activator of NF-κB Signaling

Activation of the DDR in response to ROS-induced DNA damage leads to the activation of ATM and ATR kinases, which phosphorylate numerous protein substrates, including histone H2AX and proteins involved in inflammatory signaling [90,91,92,93,94]. One key effector of this response is the activation of NF-κB, a master regulator of proinflammatory, adhesion, and prosurvival gene expression. Mechanistically, ATM kinase activated in the nucleus can initiate a signaling cascade leading to the phosphorylation and degradation of the inhibitor IκB, enabling NF-κB translocation to the nucleus [90,91,92,93,94]. Alternatively, ATM may act in the cytoplasm, where it interacts with the IκB kinase complex, directly modulating NF-κB activity [90,91,92,93,94]. This DDR-dependent NF-κB activation has been described in response to various genotoxic stressors and is considered a mechanism linking DNA damage to inflammation [90,91,92,93,94]. In aortopathies, chronic DDR activation may therefore lead to persistent NF-κB upregulation in vascular smooth muscle cells and endothelial cells, resulting in increased expression of proinflammatory cytokines such as IL-6, TNF-α, and MCP-1 [90,91,92,93,94,95,96,97]. This condition promotes the recruitment of inflammatory cells to the aortic wall, enhancing vascular tissue remodeling and degeneration [90,91,92,93,94,95,96,97,98].

### 4.2. PARP1-Dependent Modulation of NF-κB Activity

*PARP1* plays a key role in the response to DNA strand breaks, but its function extends beyond genome repair. PARP1 is a potent modulator of NF-κB activity, acting as both a transcriptional coactivator and a chromatin regulator [82,100,101]. Activation of PARP1 in response to DNA damage leads to poly(ADP-ribosylation) of histone and non-histone proteins, which facilitates NF-κB access to inflammatory gene promoters. Under conditions of chronic oxidative stress, sustained PARP1 activity is observed, promoting long-term NF-κB activation and perpetuating inflammation [82,100,101,102]. In models of vascular disease, pharmacological inhibition of PARP1 led to reduced expression of inflammatory mediators and reduced vessel wall damage, suggesting a significant involvement of the PARP1–NF-κB axis in the pathogenesis of aortopathy [82,100,101,102,103].

### 4.3. Cytoplasmic DNA and Activation of the cGAS–STING Pathway

One of the key discoveries in recent years is the role of cytoplasmic DNA as a signal activating the innate immune response. DNA damage and disruption of nuclear integrity can lead to the formation of micronuclei and the release of DNA fragments into the cytoplasm [104]. These structures are recognized by cyclic GMP–AMP synthase (cGAS), an enzyme that catalyzes the production of the second messenger cGAMP, which activates the STING (Stimulator of Interferon Genes) adaptor located in the endoplasmic reticulum. Activation of the cGAS–STING pathway leads to the phosphorylation of IRF3 and NF-κB, inducing the expression of type I interferons and numerous proinflammatory genes [104]. In the context of aortopathy, oxidative stress and DNA repair dysfunction may promote the accumulation of cytoplasmic DNA in VSMCs and endothelial cells, activating the innate inflammatory response independently of pathogens [104].

### 4.4. DDR-Driven cGAS–STING Activation in Vascular Cells

Accumulating evidence indicates that chronic DDR activation and ineffective DNA repair promote cGAS–STING activation through increased micronuclei and DNA fragmentation in the cytoplasm [105]. In vascular cells, this mechanism can lead to a persistent inflammatory response, even in the absence of classical immune stimuli. Models of cellular senescence have shown that senescent cells are characterized by enhanced cGAS–STING signaling, which drives the senescence-associated secretory phenotype (SASP) [105]. Because VSMC senescence is a crucial element in the pathogenesis of aortopathy, cGAS–STING activation may constitute an important link between DDR, cellular senescence, and aortic wall inflammation [105].

### 4.5. Integrated Model of DDR–NF-κB–cGAS/STING Crosstalk in Aortopathies

Based on the available data, a model can suggest which chronic oxidative stress leads to the accumulation of DNA damage in aortic cells, activating DDR. DDR then triggers the NF-κB pathway and promotes the generation of cytoplasmic DNA, which activates cGAS-STING [89,92,106,107,108,109,110,111,112]. These parallel but mutually reinforcing pathways lead to increased expression of inflammatory mediators, recruitment of immune cells, and progression of aortic wall degeneration. This feedback loop between DNA damage and inflammation may explain why aortopathies are characterized by chronic, low-grade inflammation and why interventions targeting only one component of this axis often prove insufficient [89,92,106,107,108,109,110,111,112]. Simultaneous modulation of DDR, NF-κB, and cGAS-STING may therefore represent a promising therapeutic strategy in the treatment of aortic diseases [89,92,106,107,108,109,110,111,112].

**V. Therapeutic perspectives: existing drugs, novel therapies, and biomarkers targeting the above pathways (Section 5).**

## 5. Therapeutic Implications and Future Perspectives

The growing understanding of the molecular mechanisms linking oxidative stress, DNA damage, the DNA Damage Response (DDR), and inflammation in aortopathies has opened new therapeutic avenues beyond classical blood pressure management and surgical interventions [113].

Conventional pharmacological approaches, including the use of non-selective antioxidants, have failed to achieve the expected clinical outcomes, highlighting the need for more precise molecular interventions targeting specific pathogenic pathways [113].

Based on these insights, several emerging therapeutic strategies can be envisioned:

- **Selective modulation of oxidative stress**—development of agents targeting specific sources of reactive oxygen species within the aortic wall, rather than employing non-specific antioxidants.
- **Targeting the DDR pathway**—inhibitors or modulators of key DDR kinases may protect endothelial and vascular smooth muscle cells from excessive DNA damage.
- **Inflammation control**—precise modulation of pro-inflammatory signaling may limit aortic pathology progression and vascular remodeling.
- **Combination therapies**—integrating novel molecular interventions with standard antihypertensive treatment and appropriate surgical procedures may enhance therapeutic efficacy and patient safety.

As research progresses, increasingly personalized treatment strategies based on the molecular profile of individual patients are becoming a tangible prospect, with the potential to significantly transform the management of aortopathies in the near future.

### 5.1. Targeting Oxidative Stress: Lessons from Antioxidant Failure

Despite strong experimental evidence implicating oxidative stress in the pathogenesis of aortopathy, clinical trials using antioxidants such as vitamins C and E have failed to demonstrate significant therapeutic benefit [113]. One possible explanation is the lack of selectivity of these compounds and their limited ability to modulate local ROS production in the aortic wall. In this context, targeting specific sources of ROS, such as NADPH oxidases (NOX), appears particularly promising. NOX inhibitors have demonstrated the ability to reduce oxidative stress, inflammation, and extracellular matrix degradation in animal models of aortic aneurysm; however, their efficacy and safety in clinical trials remain the subject of further investigation [113].

### 5.2. Modulation of DNA Repair Pathways as a Therapeutic Strategy

Targeting DNA repair mechanisms represents a promising, yet still underexplored, therapeutic strategy in aortopathies. Enhancing the BER pathway, particularly through modulation of proteins such as *OGG1* or *APE1*, could limit the accumulation of oxidative DNA damage and reduce DDR activation and inflammatory processes. On the other hand, pharmacological inhibition of overactivated DDR components, such as PARP1, may be beneficial by limiting chronic inflammation and cellular dysfunction. PARP inhibitors, widely used in oncology, have demonstrated anti-inflammatory and protective effects in models of cardiovascular disease, suggesting their potential use in aortic disease. However, the long-term effects of modulating DNA repair in vascular cells require careful evaluation to avoid induction of genomic instability [114].

### 5.3. Targeting DDR–NF-κB–cGAS/STING Axis

The identification of the DDR–NF-κB–cGAS/STING axis as a key mechanism driving chronic inflammation in aortopathies opens new avenues for therapeutic interventions. Inhibiting excessive NF-κB activation can limit the expression of proinflammatory cytokines and ECM-degrading proteases, but nonselective blockade of this pathway is associated with the risk of immunosuppression. An alternative approach is selective modulation of cGAS–STING, which can limit cytoplasmic DNA-induced inflammation without affecting the physiological immune response. STING inhibitors and cGAS signal modulators are being intensively studied in the context of autoimmune and inflammatory diseases, and their potential application in aortopathies represents a promising direction for future translational research [115].

#### 5.3.1. ECM Degradation in Aortopathy: Elastin, Collagen, MMP-2/MMP-9 and TIMP Regulation

Extracellular matrix (ECM) degradation is a central feature of aortic aneurysm and dissection pathology, critically affecting the mechanical integrity of the aortic wall. The medial layer of the aorta relies on a balanced architecture composed primarily of elastin and collagen fibers, produced by vascular smooth muscle cells (VSMCs), which provide elasticity and tensile strength, respectively. Disruption of this balance—with excessive proteolysis of these ECM components—weakens the aortic wall, predisposing it to dilation, aneurysm formation, and eventual dissection or rupture. Elastin and collagen degradation in aortic tissue is principally mediated by matrix metalloproteinases (MMPs), a family of zinc-dependent endopeptidases with broad substrate specificity, and is normally restrained by their endogenous regulators, the tissue inhibitors of metalloproteinases (TIMPs) [116,117,118,119].

In aortopathic conditions such as thoracic aortic aneurysms (TAAs), multiple studies have demonstrated elevated expression and activity of *MMP-9* and, in some contexts, altered levels of *MMP-2*, indicating enhanced proteolytic potential against ECM proteins. A meta-analysis comparing TAA tissue to controls showed a significant increase in MMP-9 and a relative decrease in *TIMP-1* and *TIMP-2*, leading to an elevated *MMP*-to-*TIMP* ratio that favors matrix degradation; *MMP-2* levels were generally unchanged in TAA overall but were increased in aneurysms associated with bicuspid aortic valves (BAV) [116,117,118,119]. Similar findings of elevated *MMP-9* relative to its inhibitors in aneurysmal aortic tissue support a proteolytic environment conducive to elastin and collagen breakdown. This imbalance between proteases and inhibitors, particularly decreased TIMPs, augments *MMP* activity and accelerates ECM dissolution, weakening the aortic media structurally and functionally [116,117,118,119].

##### Elastin

Elastin, a highly elastic component critical for the recoil and resilience of the aortic wall, is particularly susceptible to degradation by matrix metalloproteinases due to their proteolytic activity. Loss of ECM integrity reduces aortic compliance, increases wall stiffness, and contributes to medial degeneration observed in aortic aneurysms [120,121,122,123,124,125]. Collagen provides tensile strength, and its degradation further undermines structural support, leading to progressive dilation and mechanical failure of the vessel wall. Alterations in the balance between elastin and collagen also increase arterial stiffness and susceptibility to damage [120,121,122,123,124,125].

##### Tissue Inhibitors of Metalloproteinases (TIMPs)

Tissue inhibitors of metalloproteinases (TIMPs)—including *TIMP-1* and *TIMP-2*—normally restrain MMP activity by forming non-covalent inhibitory complexes, thus protecting ECM components from excessive degradation. In aortopathy, diminished *TIMP* expression (particularly *TIMP-1* and *TIMP-2*) has been frequently observed alongside elevated *MMP* levels; this further skews the proteolytic balance toward ECM breakdown. The altered *MMP/TIMP* ratio is a key pathophysiological mechanism that undermines ECM integrity and promotes aneurysm progression [126,127,128,129,130]. In chronic thoracic dissection tissue, increased levels of active *MMP-9* accompanied by decreased TIMP expression create a protease-rich environment that facilitates ongoing collagen destruction, which is tightly linked to medial degeneration after dissection [126,127,128,129,130].

In summary, ECM degradation in aortopathy involves complex protease dynamics, where elevated activity of *MMPs*, particularly *MMP-2* and *MMP-9*, combined with reduced *TIMP* regulation, disrupts the structural framework of the aortic wall. This imbalance compromises the mechanical integrity of the vessel and increases the risk of aneurysm development, progression, and rupture. Such dysregulation of ECM turnover is a key feature of the molecular pathology of thoracic and abdominal aortic aneurysms and has been confirmed by histological, biochemical, and clinical studies [131].

#### 5.3.2. Vascular Smooth Muscle Cell Dysfunction and Apoptosis in Aortopathy

Vascular smooth muscle cells (VSMCs) are key regulators of aortic wall integrity, responsible for maintaining ECM homeostasis through the synthesis of structural matrix components. In aortopathy, including thoracic aortic aneurysm (TAA) and aortic dissection, VSMC dysfunction and apoptosis are central mechanisms contributing to medial degeneration, weakening of the aortic wall, and increased susceptibility to dilation and rupture [132,133,134].

##### VSMC Dysfunction

In aortopathies, vascular smooth muscle cell (VSMC) dysfunction extends beyond the oxidative stress-induced phenotypic switch described in Section 2. VSMC abnormalities are closely linked to genetic mutations (e.g., *FBN1*, *NOTCH1*, *SMAD6*) and dysregulated signaling pathways, including TGF-β, *NOTCH*, and Angiotensin II, which collectively alter proliferation, differentiation, and extracellular matrix (ECM) synthesis [130,131,132,133,134,135,136].

These molecular perturbations contribute to structural weakening of the aortic media by disrupting ECM homeostasis. Importantly, VSMC dysfunction in this context is not only a response to oxidative damage but also a primary driver of medial layer instability, thereby influencing susceptibility to aneurysm formation and progression [130,131,132,133,134,135].

Moreover, VSMCs with impaired signaling exhibit enhanced pro-inflammatory and pro-remodeling secretory activity, exacerbating vascular inflammation and accelerating pathological aortic remodeling. This underscores their central role as both effectors and amplifiers of aortic wall degeneration, distinct from oxidative stress-induced mechanisms [130,131,132,133,134,135,136].

##### VSMC Apoptosis

Apoptosis of VSMCs is a hallmark of medial degeneration in aortopathy. Loss of these cells diminishes the population responsible for maintaining ECM integrity, impairing the repair of structural components and contributing to medial weakening. Mechanistically, apoptosis can be triggered by oxidative stress, inflammatory cytokines, dysregulated TGF-β signaling, or mechanical stress due to abnormal hemodynamics. Studies of aneurysmal tissue have demonstrated increased VSMC apoptosis in both sporadic and genetically associated TAA, correlating with reduced wall strength. Furthermore, VSMC apoptosis can create a positive feedback loop, as the loss of cells exacerbates ECM instability, which in turn increases wall stress and promotes further cell death [137,138,139,140].

##### Clinical Relevance

The combination of VSMC dysfunction and apoptosis underlies the progression of aortopathies. Medial degeneration, characterized by loss of VSMCs and disruption of ECM integrity, is observed in both syndromic (e.g., Marfan syndrome) and nonsyndromic TAAs. Therapeutic approaches aimed at preserving VSMC viability or modulating their phenotype, such as angiotensin II receptor blockers or TGF-β pathway modulators, are under investigation to slow aneurysm progression [141,142,143,144,145].

#### 5.3.3. The Role of ROS in the Vascular Smooth Muscle Cells (VSMCs), Endothelial Cells, and Extracellular Matrix (ECM) Components

Oxidative stress, resulting from an imbalance between reactive oxygen species (ROS) production and antioxidant defense mechanisms, plays a critical role in the pathogenesis of aortic aneurysms and dissections. Elevated ROS levels induce cellular damage in vascular smooth muscle cells (VSMCs), endothelial cells, and extracellular matrix (ECM) components, contributing to medial degeneration and weakening of the aortic wall [146,147,148,149,150].

##### Sources of ROS in the Aortic Wall

While Section 2 comprehensively describes ROS production in the aortic wall, this section emphasizes context-specific modulators of ROS generation relevant to aortopathy progression.

Angiotensin II (Ang II) signaling is a major driver of ROS amplification in VSMCs, linking hemodynamic stress to vascular remodeling. In patients with bicuspid aortic valve (BAV)-associated aortopathy, mechanical factors such as altered flow patterns and increased shear stress further exacerbate ROS-mediated vascular injury. These stimuli act in synergy with predisposing genetic and molecular factors to amplify oxidative damage, accelerating aortic dilation and predisposition to dissection [151,152,153,154].

Additionally, crosstalk between inflammatory cells and the aortic wall microenvironment potentiates ROS production, not merely as a source of oxidative damage but as a modulator of disease-specific signaling, including VSMC senescence, matrix remodeling, and endothelial dysfunction [151,152,153,154]. This perspective highlights ROS as a pathological amplifier rather than the primary initiating factor, complementing the mechanistic details provided in Section 2.

##### Effects of ROS on VSMCs and ECM

Excessive ROS contributes to VSMC dysfunction and apoptosis, reducing the cellular capacity to maintain ECM integrity. ROS can directly modify ECM components, impairing their mechanical properties, and can activate matrix metalloproteinases, enhancing ECM degradation. This establishes a feed-forward loop in which oxidative stress accelerates ECM destabilization, further compromising aortic wall integrity [2,5,155,156,157,158].

##### ROS and Signaling Pathways

ROS modulate several signaling pathways implicated in aortopathy. For example, oxidative stress can dysregulate TGF-β signaling, promoting excessive ECM remodeling, and influence *NOTCH1* activity, affecting VSMC proliferation and differentiation. Additionally, ROS amplify Ang II-mediated vascular inflammation, VSMC apoptosis, and ECM breakdown, collectively accelerating aneurysm progression [158,159,160,161,162].

##### Clinical Implications

Markers of oxidative stress, including lipid peroxidation products and oxidized proteins, are elevated in aneurysmal aortic tissue. Therapeutic strategies targeting ROS, such as antioxidants, NADPH oxidase inhibitors, or Ang II receptor blockers, have shown promise in preclinical models reducing aortic dilation and medial degeneration [158,163,164].

### 5.4. Inflammation in Aortopathy: IL-6, TNF-α, and Immune Signaling Pathways

Inflammation plays a central role in the pathogenesis and progression of aortic aneurysms and dissections. Both innate and adaptive immune responses contribute to medial degeneration, extracellular matrix (ECM) degradation, vascular smooth muscle cell (VSMC) dysfunction, and overall weakening of the aortic wall [165,166].

#### 5.4.1. Pro-Inflammatory Cytokines: IL-6 and TNF-α

Elevated levels of cytokines such as interleukin-6 (IL-6) and tumor necrosis factor-alpha (TNF-α) have been consistently observed in aneurysmal aortic tissue. IL-6 promotes VSMC from a contractile to a synthetic state, induces expression of matrix metalloproteinases (MMP-2 and MMP-9), and enhances inflammatory cell recruitment, thereby contributing to ECM breakdown and aortic wall weakening [167,168]. TNF-α exacerbates vascular inflammation by activating NF-κB signaling, promoting VSMC apoptosis, and stimulating oxidative stress, which further amplifies ECM degradation and medial degeneration [165,166,167,168].

#### 5.4.2. Immune Cell Infiltration and Signaling

In aortopathy, the aortic media and adventitia are infiltrated by macrophages, T lymphocytes, and mast cells, which produce cytokines, chemokines, and reactive oxygen species (ROS). Activated macrophages release MMPs, while T cells contribute to chronic inflammation through cytokine secretion. Pattern recognition receptor signaling (e.g., TLR2/4) and NF-κB activation are key immune pathways mediating the inflammatory response in the aortic wall [169,170]. Additionally, pro-inflammatory cytokines interact with other pathways such as TGF-β, *NOTCH1*, and Ang II, creating a feed-forward loop that promotes ECM degradation, VSMC apoptosis, and oxidative stress, all contributing to aneurysm progression [169,170,171].

#### 5.4.3. Clinical Implications

Circulating levels of IL-6 and TNF-α are elevated in patients with thoracic and abdominal aortic aneurysms and correlate with aneurysm size and growth rate, suggesting their potential as biomarkers for disease progression. Targeting inflammatory pathways, for example, via anti-TNF therapy or IL-6 receptor blockade, is being explored in experimental models as a strategy to slow aneurysm development and medial degeneration [172,173,174,175].

### 5.5. MicroRNAs and Epigenetic Regulation in Aortopathy

MicroRNAs (miRNAs) and epigenetic modifications play crucial roles in the regulation of gene expression involved in aortic wall homeostasis, extracellular matrix (ECM) integrity, vascular smooth muscle cell (VSMC) function, and inflammation, all of which contribute to the pathogenesis of aortic aneurysms and dissections.

#### 5.5.1. MicroRNAs (miRNAs)

miRNAs are short non-coding RNAs that post-transcriptionally regulate gene expression by binding to target mRNAs, leading to their degradation or translational repression. In aortopathy, several miRNAs have been implicated in key pathological processes:

- miR-29 family (miR-29a/b/c): Upregulated in aneurysmal tissue, these miRNAs suppress the expression of ECM components, promoting medial degeneration and weakening of the aortic wall [176,177].
- miR-21: Regulates VSMC apoptosis and proliferation; its dysregulation contributes to VSMC loss and aortic wall remodeling [178].
- miR-145 and miR-143: Maintain the contractile phenotype of VSMCs. Downregulation in aneurysmal tissue leads to VSMC phenotypic switching from contractile to synthetic phenotype, increasing ECM degradation and inflammation [179].
- miR-133 and miR-30: Modulate MMP expression and TGF-β signaling, linking miRNA dysregulation to ECM remodeling and aortic dilation [180,181].

#### 5.5.2. Epigenetic Mechanisms

Epigenetic modifications, including DNA methylation, histone modifications, and chromatin remodeling, regulate the transcriptional landscape of genes involved in aortic structure and function. In aortopathy:

- DNA methylation: Hypermethylation or hypomethylation of promoters of ECM and VSMC-related genes alters their expression, contributing to structural instability [182].
- Histone modifications: Alterations in histone acetylation and methylation affect genes regulating inflammation, VSMC differentiation, and ECM homeostasis, facilitating aneurysm progression [183].
- Interaction with signaling pathways: Epigenetic changes can modulate TGF-β, *NOTCH*, and Ang II signaling, amplifying medial degeneration, oxidative stress, and inflammation in the aortic wall [184].

#### 5.5.3. Clinical Implications

miRNAs and epigenetic marks represent potential biomarkers for early detection of aneurysm formation and progression. Therapeutic targeting of specific miRNAs (e.g., antagomiRs or miRNA mimics) and epigenetic modifiers (e.g., HDAC inhibitors) is being investigated in preclinical models to stabilize aortic structure and slow aneurysm growth [176,177,178,179,180,181,182,183,184].

## 6. Hemodynamics and ECM Degradation in Aortopathy

Hemodynamic forces, including wall shear stress, cyclic stretch, and turbulent flow, play a critical role in maintaining the structural integrity of the aortic wall and modulating extracellular matrix (ECM) remodeling. Abnormal hemodynamics, often associated with bicuspid aortic valve (BAV), aortic dilation, or hypertension, contribute to medial degeneration, ECM degradation, and aneurysm formation [185,186].

### 6.1. Impact of Shear Stress and Flow Patterns

Regions of the aorta exposed to disturbed or oscillatory flow exhibit reduced endothelial nitric oxide (NO) production, increased oxidative stress, and elevated expression of inflammatory cytokines such as IL-6 and TNF-α, which together promote ECM breakdown [3,4]. Low or oscillatory wall shear stress also activates matrix metalloproteinases in VSMCs and the adventitial layer, leading to ECM degradation and weakening of the aortic media [187,188].

#### 6.1.1. Cyclic Stretch and VSMC Response

Abnormal mechanical stretch, resulting from increased blood pressure or altered aortic geometry, induces VSMC dysfunction from contractile to synthetic states. This is accompanied by increased MMP secretion, reduced TIMP expression, and heightened oxidative stress, collectively accelerating ECM degradation [17,189]. VSMC apoptosis triggered by mechanical stress further compromises the repair capacity of the aortic wall.

#### 6.1.2. Flow-Induced Molecular Signaling

Hemodynamic forces modulate several key signaling pathways in the aortic wall. Laminar shear stress maintains *NOTCH1* activity and VSMC homeostasis, while disturbed flow reduces NOTCH1 signaling, enhancing susceptibility to ECM degradation [7]. Similarly, TGF-β signaling is activated by abnormal stretch and shear stress, driving pathological remodeling of elastin and collagen networks [190]. Angiotensin II-mediated responses are also amplified under altered hemodynamic conditions, further promoting inflammation, oxidative stress, and ECM breakdown [191].

#### 6.1.3. Clinical Implications

**Figure 5:**
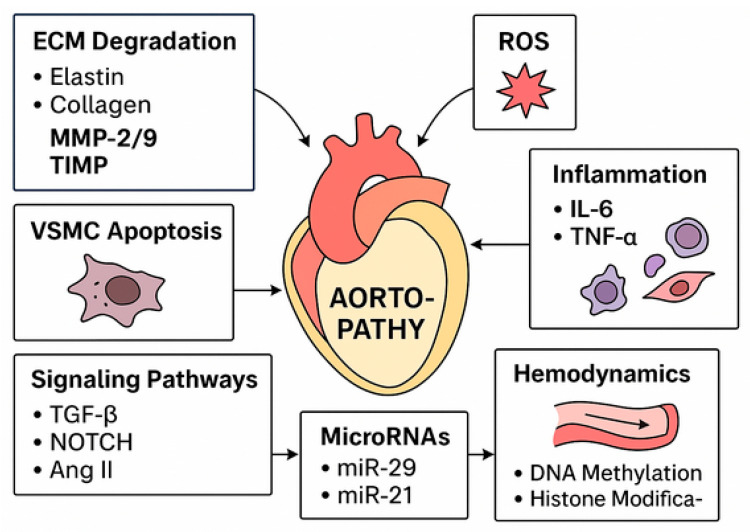
Integrated molecular, cellular, and biomechanical mechanisms contributing to the development and progression of aortopathy.

Hemodynamic abnormalities contribute to the regional susceptibility of the ascending aorta to aneurysm formation, particularly in patients with BAV, where asymmetric flow patterns create focal stress zones. Computational fluid dynamics (CFD) studies have demonstrated that areas of high oscillatory shear correlate with medial degeneration and enhanced ECM turnover, highlighting the interplay between mechanical forces and molecular pathology in aortopathy [17,185,186,187,188,189,190,191] (Figure 5).

The figure summarizes the complex interplay of molecular, cellular, and hemodynamic factors that collectively drive the pathogenesis of aortopathy. Central to this process is the progressive weakening and remodeling of the aortic wall, resulting from coordinated alterations in extracellular matrix (ECM) homeostasis, vascular smooth muscle cell (VSMC) function, inflammatory signaling, oxidative stress, and epigenetic regulation.

Excessive degradation of ECM components, including elastin and collagen, is mediated by increased activity of matrix metalloproteinases (*MMP-2* and *MMP-9*) and insufficient counter-regulation by tissue inhibitors of metalloproteinases (TIMPs), leading to loss of structural integrity and elastic recoil of the aortic wall. Concurrently, enhanced apoptosis of VSMCs reduces cellular density within the medial layer, further compromising mechanical strength and impairing adaptive repair mechanisms.

Oxidative stress, reflected by elevated levels of reactive oxygen species (ROS), amplifies inflammatory responses and promotes cellular damage, while chronic inflammation, characterized by increased expression of pro-inflammatory cytokines such as interleukin-6 (IL-6) and tumor necrosis factor-α (TNF-α), accelerates ECM remodeling and VSMC phenotypic switching from contractile to synthetic phenotype. Dysregulation of key signaling pathways, including transforming growth factor-β (TGF-β), *NOTCH*, and angiotensin II (Ang II), integrates mechanical and biochemical stimuli, modulating gene expression patterns that govern cell survival, differentiation, and matrix turnover.

Post-transcriptional regulation by microRNAs, notably miR-29 and miR-21, further fine-tunes ECM synthesis and degradation, as well as VSMC behavior. In parallel, epigenetic mechanisms, such as DNA methylation and histone modifications, contribute to long-term reprogramming of vascular cells, sustaining maladaptive remodeling responses. Altered hemodynamic forces, including abnormal wall shear stress and cyclic strain, serve as upstream triggers that initiate and perpetuate these molecular cascades.

Together, these interconnected processes converge to produce progressive aortic dilation, increased wall stiffness, and heightened susceptibility to aneurysm formation and dissection. Arrows indicate the dynamic interactions and feedback loops among molecular, cellular, and biomechanical factors that collectively shape the development and progression of aortopathy.

#### 6.1.4. Targeting Cellular Senescence and SASP

Cellular senescence, a consequence of chronic DDR activation and oxidative stress, plays a significant role in aortic wall degeneration. Senescent cells secrete numerous inflammatory and proteolytic mediators within the SASP, which enhance vascular remodeling and structural weakening of the aorta [21]. In this context, senolytics, drugs that selectively eliminate senescent cells, and senomorphs, which modulate the SASP phenotype, represent a new class of potential therapies. Preliminary studies indicate that eliminating senescent cells can improve vascular function and reduce inflammation; however, the application of these strategies in aortopathies requires further preclinical and clinical studies [21].

##### Integrated Pathophysiology and Clinical Implications of Aortopathy

Comprehensive Overview of Molecular, Cellular, and Hemodynamic Mechanisms in Aortopathy

Aortopathy, including thoracic and abdominal aortic aneurysms, is a multifactorial disease resulting from the complex interplay of genetic predisposition, molecular signaling dysregulation, cellular dysfunction, extracellular matrix (ECM) degradation, oxidative stress, inflammation, epigenetic alterations, microRNA (miRNA) dysregulation, and abnormal hemodynamics. Understanding these mechanisms is critical for elucidating disease pathogenesis and developing targeted therapeutic strategies.

##### Extracellular Matrix (ECM) Degradation

The integrity of the aortic wall relies on a balanced ECM, which provides elasticity and tensile strength. Dysregulated matrix metalloproteinases and reduced tissue inhibitors of metalloproteinases (TIMPs) promote excessive ECM degradation, weakening the medial layer and facilitating aneurysm formation. Genetic mutations (e.g., FBN1, SMAD6) and signaling dysregulation contribute to this imbalance, further exacerbating ECM instability [1,2,3,4].

##### Vascular Smooth Muscle Cell (VSMC) Dysfunction and Apoptosis

VSMCs are essential for ECM maintenance and aortic wall homeostasis. In aortopathy, VSMCs undergo phenotypic switching from a contractile to a synthetic state, secrete MMPs, and produce pro-inflammatory cytokines. Enhanced apoptosis of VSMCs, induced by oxidative stress, mechanical stress, or inflammatory signaling, reduces repair capacity of the aortic wall, exacerbating medial degeneration [5,6,7,8].

##### Oxidative Stress and Reactive Oxygen Species (ROS)

Excessive ROS generation from NADPH oxidases, mitochondria, xanthine oxidase, and uncoupled eNOS, often amplified by Angiotensin II (Ang II) signaling, contributes to VSMC apoptosis, ECM protein oxidation, and MMP activation. ROS interact with TGF-β and *NOTCH1* signaling, further promoting medial degeneration and aneurysm progression [9,10,11,12].

##### Inflammation

Chronic vascular inflammation is mediated by innate and adaptive immune cells, including macrophages, T lymphocytes, and mast cells, which secrete cytokines such as IL-6 and TNF-α. These mediators activate NF-κB and Toll-like receptor pathways, stimulating MMP expression, oxidative stress, and VSMC apoptosis, thereby accelerating ECM degradation and aneurysm expansion [13,14,15,16,17]. Recent evidence also implicates the cGAS–STING pathway as a key molecular link between DNA damage, oxidative stress, and sterile vascular inflammation. Activation of cGAS–STING in vascular cells promotes type I interferon signaling and proinflammatory cytokine production, reinforcing NF-κB-mediated responses and sustaining chronic vascular remodeling. Incorporating this pathway provides a more comprehensive view of inflammatory signaling in aortopathies and highlights potential molecular targets for intervention [82,90,91,92,93,94,98,104,112,115].

##### Molecular Signaling Pathways

- TGF-β signaling is critical for ECM synthesis and VSMC differentiation; dysregulation due to genetic or environmental factors leads to excessive ECM remodeling and medial degeneration.
- *NOTCH1* signaling regulates VSMC proliferation, differentiation, and endothelial homeostasis; reduced *NOTCH1* activity enhances susceptibility to aneurysm formation.
- Ang II signaling promotes VSMC proliferation, oxidative stress, inflammation, and ECM degradation, interacting with TGF-β and *NOTCH* pathways to amplify aneurysm pathology [6,18,19,20].

##### MicroRNAs and Epigenetics

miRNAs such as miR-29, miR-21, miR-145, and miR-143 regulate ECM protein expression, VSMC phenotype, apoptosis, and inflammatory responses. Dysregulation of these miRNAs contributes to medial degeneration and aortic dilation. Epigenetic modifications, including DNA methylation and histone alterations, modulate transcription of ECM-related genes, VSMC differentiation, and inflammatory mediators, and interact with signalling pathways like TGF-β and *NOTCH* to exacerbate aneurysm progression.

##### Hemodynamic Forces

Abnormal hemodynamics, including disturbed flow, low or oscillatory shear stress, and increased cyclic stretch, contribute to regional susceptibility of the aorta to aneurysm formation. These forces impair endothelial function, activate VSMC MMP secretion, increase oxidative stress, and modulate signaling pathways (TGF-β, *NOTCH1*, Ang II), resulting in localized ECM degradation and medial weakening.

##### Integrated Pathophysiology

Aortopathy represents a convergence of genetic, molecular, cellular, and mechanical factors. Mutations in structural (*FBN1*) or signaling (*NOTCH1*, *SMAD6*) genes disrupt ECM homeostasis, while dysregulated signaling pathways (TGF-β, *NOTCH1*, Ang II) and epigenetic changes amplify VSMC dysfunction, apoptosis, oxidative stress, and inflammation. Hemodynamic stress further localizes injury and ECM breakdown, leading to progressive dilation, aneurysm formation, and risk of dissection or rupture.

##### Clinical Implications

Understanding the multifactorial mechanisms underlying aortopathy provides opportunities for biomarker development (e.g., circulating miRNAs, inflammatory cytokines) and targeted therapies aimed at stabilizing the aortic wall and preventing aneurysm progression. Current and emerging therapeutic strategies include angiotensin II receptor blockers, TGF-β modulators, antioxidant approaches, anti-inflammatory interventions, miRNA-based therapeutics, and novel interventions targeting the cGAS–STING pathway, such as STING inhibitors or modulators, which may help mitigate sterile vascular inflammation and DNA damage-driven vascular remodeling. Incorporating these approaches reflects the latest advances in the field and expands the therapeutic landscape for aortic disease [17,21,36,48,104,112,115,145,161,170,184,185,186,187,188,189,190,191,192,193].

#### 6.1.5. Future Perspectives, Challenges, and Limitations

Despite substantial progress in elucidating the molecular mechanisms underlying aortopathy, many fundamental questions remain unanswered [3,21]. One of the major challenges is the identification of reliable biomarkers of DNA damage and DNA damage response (DDR) activation that could enable early diagnosis, monitoring of disease progression, and evaluation of therapeutic efficacy. Promising candidates include oxidative DNA damage markers such as 8-oxo-dG, indicators of double-strand breaks including γ-H2AX, PARP1 activity, and markers of innate immune activation mediated by the cGAS–STING pathway [84,88,104,112,115]. However, their clinical applicability requires validation in large, well-characterized patient cohorts and methodological standardization across laboratories [3,21].

A deeper understanding of the distinct molecular mechanisms driving degenerative versus genetically determined aortopathies also represents a key research priority. While genetic forms of the disease are frequently linked to mutations affecting extracellular matrix organization, TGF-β signaling, and vascular smooth muscle cell (VSMC) function, degenerative aortopathies appear to be predominantly driven by cumulative oxidative stress, chronic inflammation, and age-associated decline in DNA repair capacity [66,67,71,72,143,144,145]. Dissecting shared and disease-specific DDR pathways may therefore facilitate the development of personalized diagnostic and therapeutic strategies.

Despite accumulating experimental evidence, the field continues to be challenged by heterogeneous and sometimes contradictory findings, which likely reflect differences in disease models, experimental conditions, patient populations, and analytical techniques [11,146,147,159]. Small sample sizes, cross-sectional study designs, and limited longitudinal data further complicate interpretation and comparison of results, hindering the establishment of robust mechanistic conclusions [1,146]. Standardized protocols, harmonized outcome measures, and large-scale multicenter studies will be essential to improve reproducibility and translational relevance.

A major limitation in current research remains the restricted translational value of animal models, which often fail to fully recapitulate the complexity of human aortic pathology. Species-specific differences in vascular architecture, hemodynamics, lifespan, and immune responses may profoundly influence disease progression and therapeutic outcomes [123,131,134]. Indeed, several therapeutic strategies that demonstrated promising results in murine models failed to show clinical benefit in human trials, highlighting the translational gap between experimental and clinical research [141,142,147]. These limitations underscore the need for improved experimental platforms, including human induced pluripotent stem cell-derived vascular cells, organ-on-chip technologies, and advanced ex vivo tissue models.

Another important challenge lies in the translation of DDR-targeted molecular mechanisms into clinically safe and effective therapies. While pharmacological modulation of DNA repair pathways, oxidative stress responses, and inflammatory signaling holds considerable promise, such interventions may entail substantial risks, including genomic instability, impaired tissue regeneration, and increased oncogenic potential [88,112,170]. A thorough understanding of dose-dependent effects, tissue specificity, and long-term safety is therefore essential before clinical implementation.

Finally, significant gaps persist in our understanding of cell type-specific DDRs within the aortic wall. Endothelial cells, vascular smooth muscle cells, fibroblasts, and infiltrating immune cells exhibit distinct DNA repair capacities and stress response pathways, yet most existing studies rely on bulk tissue analyses that obscure this heterogeneity [23,24,84,89]. Emerging single-cell and spatial multi-omics approaches offer powerful tools to resolve these complexities and identify precise cellular drivers of disease initiation and progression, thereby enabling more targeted therapeutic strategies.

In summary, addressing these challenges through integrative molecular, genomic, and clinical approaches will be crucial for advancing personalized medicine in aortopathy. A comprehensive understanding of DDR dynamics across cell types and disease subtypes, combined with validated biomarkers and improved translational models, will be essential to bridge the gap between mechanistic discovery and clinical intervention [3,21].

#### 6.1.6. Concluding Remarks

In summary, the molecular mechanisms discussed in this section highlight the critical contribution of oxidative stress, DNA damage, and dysregulated DNA damage response (DDR) pathways to the development and progression of aortopathy [1,5,11,12]. Accumulating evidence indicates that persistent genomic instability and chronic activation of inflammatory signaling cascades, particularly those mediated by the cGAS–STING axis, represent central drivers of vascular remodeling, smooth muscle cell dysfunction, and extracellular matrix degradation [89,104,109,110,111,112].

Targeting these interconnected pathways may offer novel therapeutic opportunities complementary to current clinical management strategies. Pharmacological modulation of oxidative stress, enhancement of DNA repair capacity, and selective inhibition of maladaptive DDR and cGAS–STING signaling may potentially attenuate inflammation, limit vascular wall degeneration, and slow disease progression [23,24,115]. However, further experimental and clinical studies are required to define the safety, specificity, and long-term efficacy of such targeted approaches in patients with aortopathy [104,112,115].

## 7. Conclusions

Aortopathies represent a heterogeneous group of vascular disorders driven by a complex interplay between genetic predisposition, molecular dysregulation, cellular dysfunction, and abnormal hemodynamic forces. Increasing evidence indicates that oxidative stress, DNA damage accumulation, defective DNA repair, and sterile vascular inflammation constitute central mechanisms linking biomechanical stress to progressive degeneration of the aortic wall. In particular, the functional crosstalk between the DNA damage response (DDR), NF-κB signaling, and the cGAS–STING pathway provides a mechanistic framework explaining persistent, sterile vascular inflammation and sustained vascular remodeling.

A deeper understanding of these integrated molecular and cellular processes may enable the identification of novel diagnostic biomarkers and therapeutic targets. Strategies aimed at modulating oxidative stress, DNA repair capacity, inflammatory signaling, and mechanotransduction may complement current surgical approaches and contribute to improved risk stratification, personalized therapy, and long-term outcomes in patients with aortic disease.

## Figures and Tables

**Table 1:** Key genes involved in thoracic aortopathy: molecular mechanisms, cellular targets, extracellular matrix (ECM) effects, signaling interactions, and clinical phenotypes.

Gene	Main Molecular Pathway	Target Cells	Effect on ECM and Vessel Wall	Key Signaling Interactions	Clinical Phenotype
NOTCH1	Notch signaling (NICD → HES/HEY)	VSMCs, endothelial cells	Reduced structural integrity, ↑ MMP-2/9, ECM degradation	TGF-β, miR-145, miR-34a	Bicuspid aortic valve, ascending aortic aneurysms, dissections
FBN1	Structural ECM protein (fibrillin-1)	VSMCs, fibroblasts	Impaired elastin architecture, abnormal microfibril assembly	TGF-β (release from ECM stores)	Marfan syndrome: ascending aneurysms, valve disease
TGFBR1/TGFBR2	TGF-β receptor signaling (SMAD2/3)	VSMCs, fibroblasts	Dysregulated ECM remodeling, ↑ MMP activity, fibrosis	SMADs, Notch	Loeys–Dietz syndrome: aggressive aneurysms, dissections
ACTA2	Smooth muscle contraction (α-actin)	VSMCs	Impaired contractility, abnormal ECM organization	Rho/ROCK, Notch (indirect)	Familial thoracic aortic aneurysms, early dissections
SMAD3	TGF-β signal transduction	VSMCs, fibroblasts	Altered collagen and elastin synthesis	TGF-β, Notch (indirect)	Aneurysmal syndrome type 1, thoracic aneurysms
MYH11	Smooth muscle contraction (β-myosin heavy chain)	VSMCs	Reduced contractility, cytoskeletal disorganization	Rho/ROCK, TGF-β (indirect)	Familial ascending aortic aneurysms, dissections

## Data Availability

No new data were created or analyzed in this study. Data sharing is not applicable to this article.

## Abbreviations

AAA	abdominal aortic aneurysm
TAA	thoracic aortic aneurysm
ROS	reactive oxygen species
RNS	reactive nitrogen species
MMPs	matrix metalloproteinases
8-oxo-dG	8-oxy-2′-deoxyguanosine
DDR	DNA Damage Response
BER	base excision repair
DSBR	double-strand break repair
VSMC	vascular smooth muscle cell
MMP-2 and MMP-9	matrix metalloproteinases
mtDNA	mitochondrial DNA
MPO	myeloperoxidase oxidase
SASP	senescence-associated secretory phenotype
(AP) sites	apurinic/apyrimidinic
*OGG1*	8-oxoguanine DNA glycosylase 1
8-oxoG	8-oxo-7,8-dihydroguanine
*APE1*	apurinic/apyrimidinic endonuclease 1
ECM	extracellular matrix
*MPG*	methylpurine DNA glycosylase
*ANPG*	O6-alkylguanine DNA alkyltransferase, also referred to as MGMT
*COX-1* and *COX-2*	Cyclooxygenases
*LOX*	Lysyl oxidase
TAAD	thoracic aortic aneurysm and dissection
NICD	intracellular domain
BAV	bicuspid aortic valve
AscAA	ascending aortic aneurysm
*FBN-1*	extracellular matrix glycoprotein fibrillin
BMP	bone morphogenetic protein
Ang II	Angiotensin II
miRNA	microRNA
cGAS	cyclic GMP–AMP synthase
cGAMP	second messenger
IκB kinase complex	Inhibitor of nuclear factor kappa-B kinase complex
STING	Stimulator of Interferon Genes
TIMPs	tissue inhibitors of metalloproteinases
